# Generative Modelling of Cortical Receptor Distributions from Cytoarchitectonic Images in the Macaque Brain

**DOI:** 10.1007/s12021-024-09673-7

**Published:** 2024-07-08

**Authors:** Ahmed Nebli, Christian Schiffer, Meiqi Niu, Nicola Palomero-Gallagher, Katrin Amunts, Timo Dickscheid

**Affiliations:** 1 Institute of Neuroscience and Medicine (INM-1), Research Centre Jülich, Jülich, Germany; 2 Helmholtz AI, Research Centre Jülich, Jülich, Germany; 3 Institute of Computer Science, Heinrich Heine University Düsseldorf, Düsseldorf, Germany; 4 Cécile & Oscar Vogt Institute for Brain Research, University Hospital Düsseldorf, Heinrich-Heine-University Düsseldorf, Düsseldorf, Germany

**Keywords:** Generative adversarial networks, Neurotransmitter receptor, Autoradiography, Conditional learning, Area-wise training

## Abstract

Neurotransmitter receptor densities are relevant for understanding the molecular architecture of brain regions. Quantitative in vitro receptor autoradiography, has been introduced to map neurotransmitter receptor distributions of brain areas. However, it is very time and cost-intensive, which makes it challenging to obtain whole-brain distributions. At the same time, high-throughput light microscopy and 3D reconstructions have enabled high-resolution brain maps capturing measures of cell density across the whole human brain. Aiming to bridge gaps in receptor measurements for building detailed whole-brain atlases, we study the feasibility of predicting realistic neurotransmitter density distributions from cell-body stainings. Specifically, we utilize conditional Generative Adversarial Networks (cGANs) to predict the density distributions of the M2 receptor of acetylcholine and the kainate receptor for glutamate in the macaque monkey’s primary visual (V1) and motor cortex (M1), based on light microscopic scans of cell-body stained sections. Our model is trained on corresponding patches from aligned consecutive sections that display cell-body and receptor distributions, ensuring a mapping between the two modalities. Evaluations of our cGANs, both qualitative and quantitative, show their capability to predict receptor densities from cell-body stained sections while maintaining cortical features such as laminar thickness and curvature. Our work underscores the feasibility of cross-modality image translation problems to address data gaps in multi-modal brain atlases.

## Introduction

Advances in imaging and analyzing large series of cell-body stained sections and neurotransmitter receptor autoradiography have significantly facilitated the study of the brain’s cytoarchitectonic, fiber, and neurochemical organization (Amunts & Zilles, 2015; Palomero-Gallagher & Zilles, 2019; Caspers et al., 2013). These techniques coupled with image analysis algorithms (Schleicher et al., 2005) capture unique attributes of different brain regions, such as laminar pattern of cortical areas, cortical thickness and neuronal density (Amunts & Zilles, 2015). In this context, brain atlases have been introduced to serve as standardized frameworks, enabling several modality measurements (e.g., cell-body stained and autoradiography) in a common reference space (Toga et al., 2006; Toga & Thompson, 1998; Amunts et al., 2020).

Whereas detailed cytoarchitectonic mapping of 248 areas in the human brain together with brain-wide measures of cell densities have already been published (Amunts et al., 2020), comprehensive measurements of neurotransmitter receptor densities by means of quantitative in vitro receptor autoradiography (Palomero-Gallagher & Zilles, 2018) are limited to a subset of 44 human cortical regions (Zilles & Palomero-Gallagher, 2017). A relatively higher amount of data is available for the macaque monkey brain, where the densities of 14 different receptor types normalized by neuronal density have been for over 100 cortical areas (Froudist-Walsh et al., 2023). Receptors are specialized proteins or protein complexes that play a crucial role in the transfer of information between neurons and are thus pivotal for understanding synaptic interactions between neurons (Palomero-Gallagher & Zilles, 2019).

At the same time, previous research of our group has shown that the distribution of receptors in the cerebral cortex and subcortical nuclei is linked to the distribution of cells in a specific manner, and, as a general rule, the localization of borders of cytoarchitectonic areas coincide with those of receptorarchitectonics (e.g., Zilles & Amunts, 2015). However, the precise relationship of cyto- and receptor distributions is a topic of intensive research (e.g., Zachlod et al., 2023). Based on such research, our work leverages the more readily available and high-resolution cell-body stained sections to impute missing or damaged receptor autoradiography sections.

Deep generative models, such as Generative Adversarial Networks (GANs) (Goodfellow et al., 2014), excel at learning patterns in data distributions to generate new, similar samples. In the realm of medical imaging, Conditional GANs (cGANs) (Mirza & Osindero, 2014) have shown particular promise (Yang et al., 2020; Armanious et al., 2020; Alotaibi, 2020; Singh & Raza, 2021). The conditional aspect allows the models to generate images based on specific medical input aspects such as the input image modality. Such conditioning makes cGANs especially useful for tasks like inter-modality image-to-image translation and medical image inpainting (Yang et al., 2020; Armanious et al., 2020). Despite this progress, the specific prediction of neurotransmitter receptor densities remains largely unexplored. The problem of missing data imputation for autoradiography has recently been addressed by Funck et al. (2022), which employs linear interpolation to estimate missing autoradiographic sections based on 3D tissue reconstructions and spatial continuity assumptions.

Unlike this prior research, our study aims to leverage cGANs for predicting local neurotransmitter receptor densities from a different imaging modality, specifically cell-body stained images. In essence, we tackle a cross-modality image translation problem to address data gaps in brain atlases. Since there are unavoidable variances in the data, such as slight morphological differences between sections, histological artifacts, and variations in radioactive labeling intensity, we choose a generative model that predicts a distribution of possible outputs instead of a direct domain translation approach which assumes a 1:1 correspondence between inputs and outputs.

We employ a cGAN architecture (Mirza & Osindero, 2014) for estimating $M_2$ and kainate receptor densities in the macaque monkey’s primary visual (V1) and primary motor (M1) cortex using cell-body stained image patches as input. This is similar to style transfer problems in computer vision (Gatys et al., 2016; Zhao, 2020). The input and corresponding target patches are derived from closely situated tissue sections, which have undergone a process of nonlinear alignment.

As it is accepted thought that distinct brain regions exhibit unique receptor balances (Palomero-Gallagher et al., 2009, 2013), one would hypothesize that informing the cGANs of brain region information (e.g., brain region label) could be crucial. To test this assumption, we propose three cGAN variants with varying conditions: 1) a set of four specialist models, each trained for a specific receptor and cortical area, 2) a set of two multi-area models, one per receptor type, trained on patches from both brain areas but explicitly conditioned by the brain area label and 3) a set of two multi-area models as in 2), but without explicit information about the brain area. Variant 3 can only rely on the information encoded in the input image patch for predicting the receptor distribution. For each model variant, we implement an identical architecture for both the generator and the discriminator. The generator and discriminator use a Convolutional Neural Network (CNN) to predict receptor patches and differentiate target receptor patches from the predicted ones, respectively.

Our study makes three key contributions:

1. We demonstrate the general ability of deep generative networks to predict realistic receptor distributions from cell-body stained input patches.
2. We show that the image patches generated by the models preserve fundamental attributes of local cortical morphology, such as cortical thickness and curvature, as encoded in the cell-body stained input patch.
3. We show that the generated density distributions for the visual area V1 and motor area M1 areas exhibit a realistic appearance without explicitly conditioning the model on specific regions of the macaque brain.

## Materials and Methods

### Multimodal Image Data of the Macaque Monkey Brain

In this study, we examined a total of 45 sections from the left hemisphere of a single macaque monkey brain, with 15 sections dedicated to each of three distinct measurements: autoradiography for cholinergic muscarinic ($M_2$) receptors, autoradiography for glutamatergic (kainate) receptors, and Nissl staining for cell body visualization. Of these, 10 sections from each measurement type were used to predict patches from the visual cortex (area V1), and the remaining 5 sections from each were used to predict patches from the motor cortex (area M1). These images were previously used to characterize the macaque monkey primary visual cortex (V1) (Rapan et al., 2022) as well as the primary motor cortex (M1) (Rapan et al., 2021). In short, unfixed shock frozen macaque monkey brains were serially sectioned in the coronal plane. The sectioning protocol was such that two consecutive sections processed for a given modality (i.e., a cell body staining or a specific receptor type) were separated by approximately 1*mm* in the brain using a light microscope (Axioplan 2 imaging, Zeiss, Germany) equipped with KS400 and Axiovision (version 4) systems. The resolution of the cell-body stained scans was $1 \mu m / \text {pixel}$.

We used images of autoradiographs encoding the distribution patterns of the $M_2$ and kainate receptors in areas V1 and M1. The experimental procedure is described in detail in the publications in which the receptors in these two areas were identified and characterized (Rapan et al., 2021, 2022). Brain sections were incubated with radiolabelled ligands selectively targeting a specific receptor type. Radiolabelled sections were then co-exposed with standards of known concentrations of radioactivity against tritium-sensitive films. The ensuing autoradiographs, which provide a visual representation of the receptor distribution, were digitized using a Charge-Coupled Device (CCD) camera (Axiocam MRm, Zeiss, Germany) and Axiovision software (Zeiss, Germany), thus enabling their densitometric analysis (Palomero-Gallagher and Zilles 2018).

**Fig. 1:**
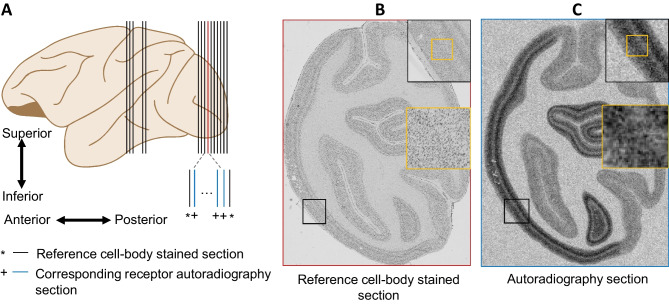
Overview of the image datasets of different modalities. **A** A schematic representation of the brain showing the locations of sections used in the study. **B** Example of a cell-body stained section from the visual cortex. **C** Example of an adjacent autoradiography section. Between each two adjacent cell-body stained sections, there are 20 sections used for different receptor autoradiographies

The obtained 8-bit images have an in-plane resolution of $20\mu m$ (Rapan et al., 2021, 2022). The grey values of the co-exposed standards are used to compute a regression curve with which a linear relationship between receptor densities (in fmol/mg protein) and the grey value in each pixel of a digitized autoradiograph can be established (Palomero-Gallagher & Zilles, 2018). These linearized images constitute the basis for the present analysis. Figure 1 provides a visual representation: Panel (A) displays a schematic of the 15 sections analyzed in this study, comprising 10 sections containing area V1 and 5 sections with area M1, corresponding to each receptor type. Panels (B) and (C) respectively showcase an overlay of cell body-stained histology and contrast-enhanced autoradiography sections.

### Extraction of Corresponding Cortical Image Patches

To train the generative models, we sample *N* corresponding pairs of image patches from both modalities $\{ (X^{\text {cyto}}_{n}, X^{\text {ar}}_{n}) \}^{N}_{n=1}$ cell-body stained and autoradiography in the cortical areas V1 and M1. The correspondence is established by image registration between an autoradiography section to its neighboring cell-body stained reference section (Fig. 1).

**Fig. 2:**
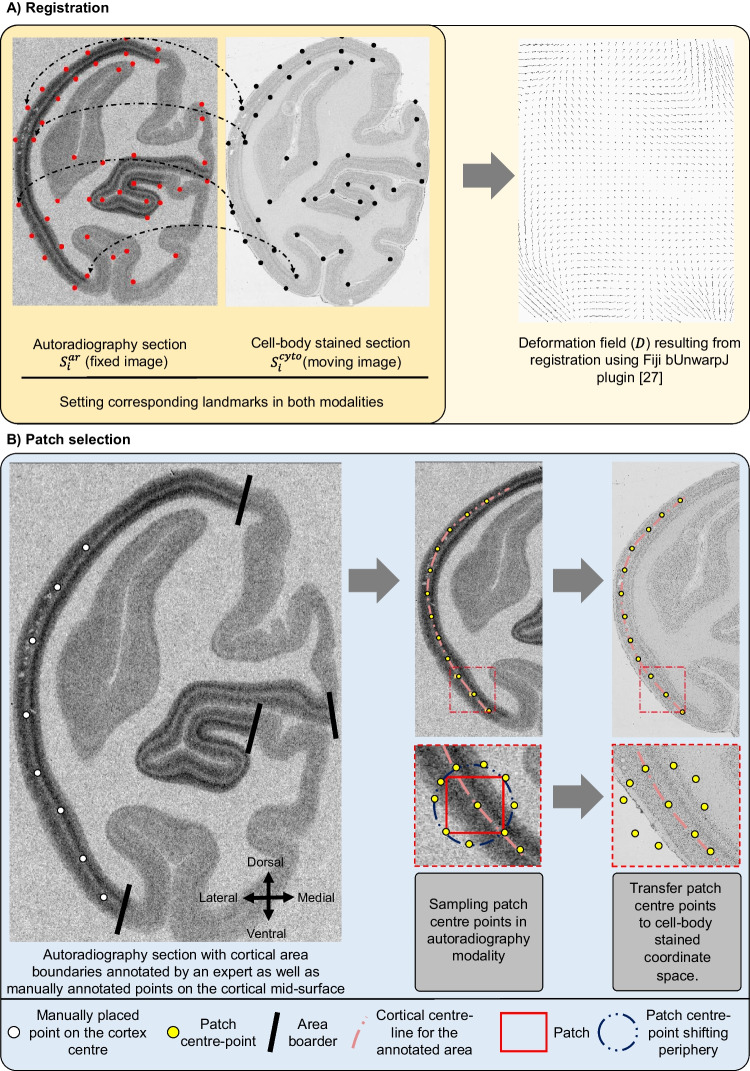
Workflow for cell-body stained and receptor autoradiography pre-processing and patch extraction. Two stages: **A** Registration: Using Fiji’s bUnwarpJ with landmark alignment, we place an average of 42 landmarks per image (A left). The strategy for placing points was to distribute them well across the cortex of the section and close to clearly visible landmarks such as the tip of a gyrus or the deepest point of a sulcus. The deformation is illustrated as a vector field (A right), where arrows denote the shift of a pixel in the moving image (cell-body stained section) to its target position in the fixed image (autoradiography section). Note that the figure shows the deformation vectors at a reduced resolution for illustration, while the actual deformation field has the same dimensions and resolution as the autoradiography section. **B** Patch Selection: patch selection: It involves curve fitting along manually identified cortical points, with equidistant points chosen as central patch locations. Around each, eight adjacent points are determined with a 0.5 mm shift. These patch center coordinates are mapped to stained sections via inverse deformation, adjusting cropping for resolution discrepancies

#### Multimodal Alignment of Neighboring Sections

The autoradiography sections are in general deformed in a nonlinear fashion relative to their corresponding cell-body stained reference sections. This is a typical, inevitable consequence of the histological processing (Fig. 1). Thus we perform a nonlinear alignment as a prerequisite for sampling corresponding patches. This is performed using the workflow illustrated Fig. 2(A).

First, reference sections are downscaled from $1 \mu m$ to $5 \mu m$ to match the resolution of the autoradiography section. Second, we identify an average of 42 anatomical landmarks per image pair, selecting them based on distinct anatomical features such as the center of specific sulci and gyri. This selection of landmark locations simplifies the validation of registration quality through visual inspection. Ten of these landmarks are randomly reserved for validation, while the remaining landmarks guide the multi-modal image registration. Third, registration is performed using the bUnwarpJ plugin in Fiji (v. 1.54f) (Arganda-Carreras et al., 2006), which applies a B-spline-based nonlinear transformation *F* to the autoradiography section (source image) to match the cell-body stained reference section (target image). The bUnwarpJ plugin uses a transformation that minimizes the Euclidean distances between the landmark pairs as well as the dissimilarity of the images as measured by the sum of squared differences. We set the relative weight between landmarks and similarity to $\lambda = 0.3$. Exact parameter settings of the plugin are provided in the supplementary materials. Finally, we apply the resulting inverse deformation field $D^{-1}$ to the held-back landmarks to visually verify the result.

#### Defining Sampling Positions for Cortical Patches

The strategy for sampling cortical image patches from the annotations is illustrated in Fig. 2(B). We first identify the midline of the cortex in the target areas by fitting a cubic spline to manually-placed control points, spaced at an average distance of $8.6\, \text {mm}$ based on empirical testing that indicated optimal curve fitting throughout the selected control points. Next, we position square patches of size $2 \times 2\, \text {mm}$ at intervals of $0.2\, \text {mm}$ along this midline. The interval size of $0.2\, \text {mm}$ was selected to ensure adequate sampling while minimizing redundancy. At each specified position $(x, y)$, we sample nine shifted patches with offsets defined by $\{(x + dx, y + dy), \forall dx, dy \in \{-0.5, 0, 0.5\} \}$. This yields an overlap rate of approximately $90\%$ which is chosen to maximize the number of training patches. The obtained patches are then mapped to the corresponding positions in the cell-body stained sections using $F^{-1}$ and $D^{-1}$. In both modalities, we place square patches of $2\, \text {mm}$ side length, using four corner points to define their boundaries. Any patches outside the sections or with tissue damage are manually excluded.

### cGAN-Based Model for Autoradiography Prediction

**Fig. 3:**
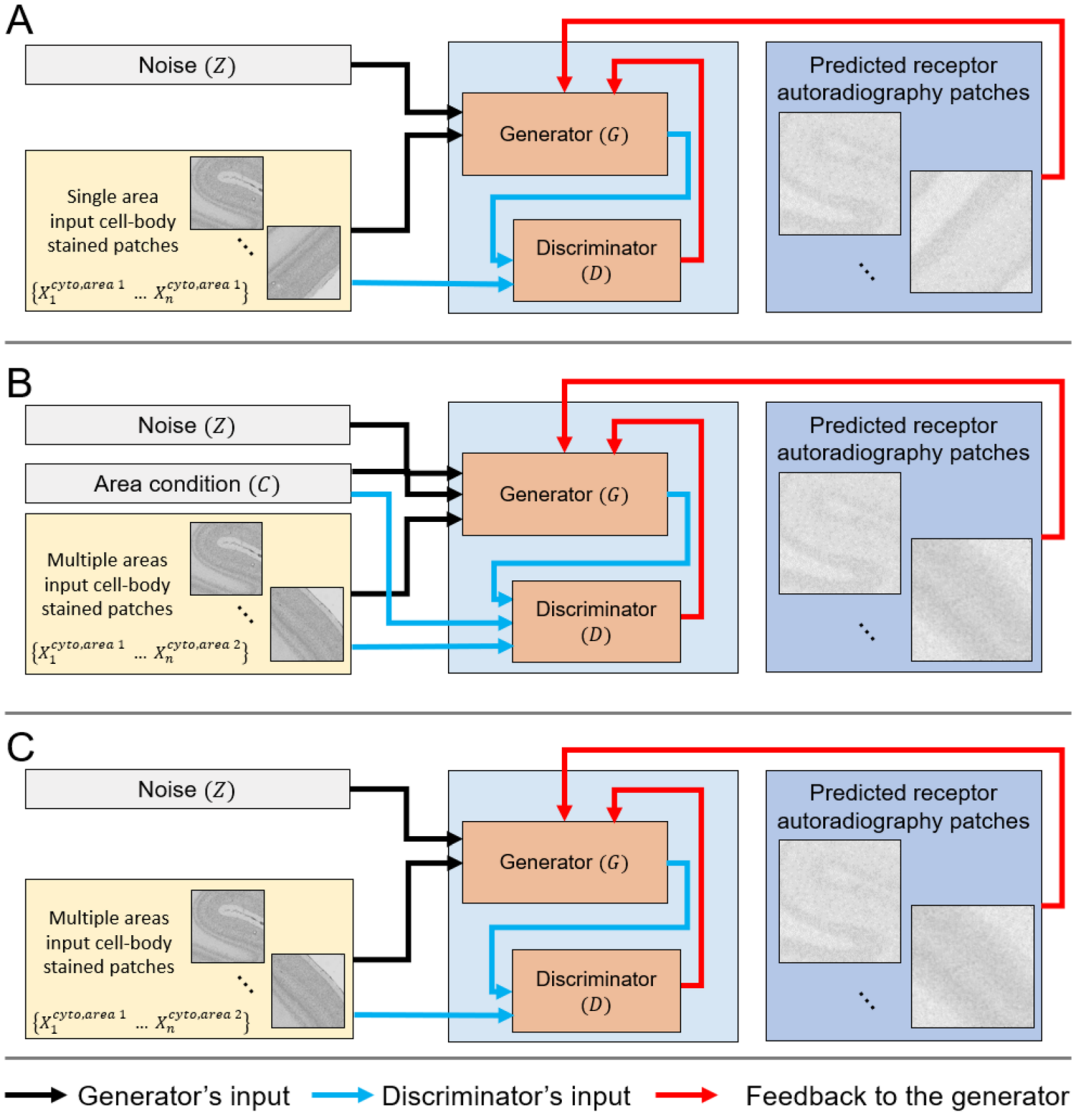
Variants of cGAN training pipelines. The figure illustrates three cGAN training models. **A** shows the Conditional single-area GAN with inputs of single-area, single-receptor cell-body stained patches and noise. **B** presents a Conditional multi-area GAN that takes multi-area patches for one receptor, noise, and a condition tensor $C$. For the conditional multi-area GAN, both generator and discriminator have an extra input channel for the area condition. **C** depicts an Unconditional multi-area GAN, similar to the Conditional single-area GAN but with multi-area for a given receptor inputs

We use three cGAN variants Fig. 3, each with different conditioning schemes for receptor prediction in macaque monkey brain regions V1 and M1. All models use the same architecture for the generator and the discriminator.

#### Model Architecture

**Fig. 4:**
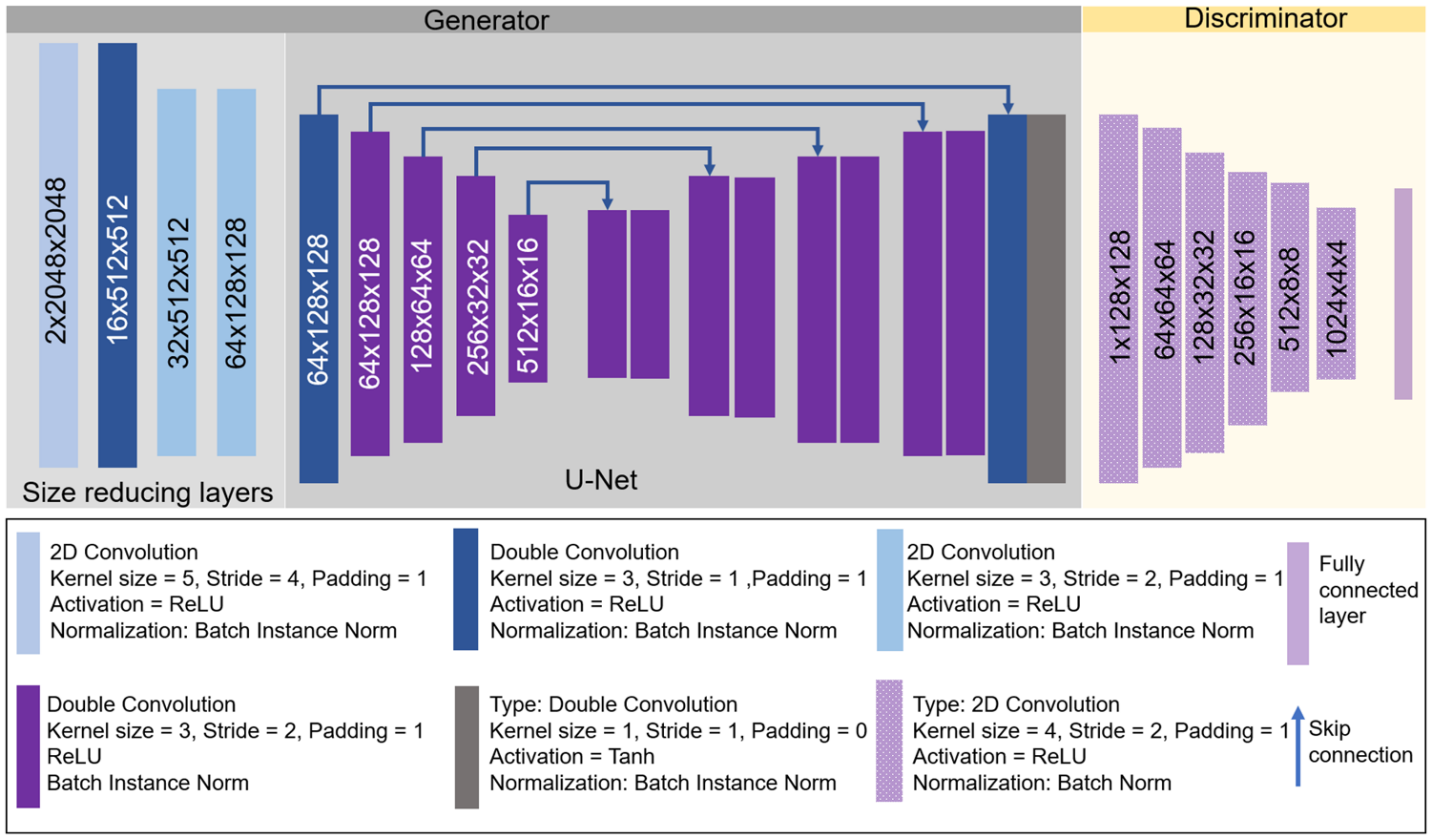
Architecture of the generator and the discriminator for the single-area GAN. The generator is composed of four convolutional layers aiming to reduce the size of the input cell-body stained histological patches to match the spatial size of the receptor patches. These four layers are followed by a UNet architecture. The discriminator is composed of five convolutional layers followed by a fully connected layer. Details of each convolutional layer could be seen in the legend

The generator $G: (X^{cyto}, Z) \rightarrow \hat{X^{ar}}$ produces samples $\hat{X^{ar}}$ conditioned on multi-channel input tensors $( X^{cyto}, Z)$. The input tensor is composed of cell-body stained patches $X^{cyto}$ with a resolution of $( 1 \mu m )$ and spatial dimensions $( 2048 \times 2048 )$ pixels, concatenated with a white noise tensor $Z$ serving as a random source. Additional input channels may be added for conditioning, depending on the model variant. Rather than merely reducing the resolution, the network aims to learn to map the input cell-body stained image patches to the output autoradiography patch distribution. The architecture comprises two main components: a backbone and a head, as shown in Fig. 4. The backbone consists of four convolutional layers that transform $X^{cyto}$ into a $( 64 \times 128 \times 128 )$ feature map and a head. The head employs an adapted U-Net architecture (Ronneberger et al., 2015) to generate samples $\hat{X^{ar}}$ with dimensions $( 128 \times 128 )$ and a resolution of $( 5 \mu m )$. All convolutional layers use Batch-Instance normalization (Nam & Kim, 2018) and ReLU activation, except for the final layer, which employs a $\tanh$ activation function. Specific layer parameters are detailed in Fig. 4.

The discriminator (*D*) distinguishes predicted patches $\hat{X^{ar}}$ from their corresponding targets $X^{ar}$. The architecture consists of four convolutional layers with kernel size 4, stride 2, and padding 1, followed by a terminal fully connected layer. Each convolutional layer is followed by a Batch Normalization layer and a LeakyReLU activation function with slope $\alpha =0.2$. Detailed parameters are given in Fig. 4.

#### Training and Loss Functions

The models are trained using the following loss function:

1 $$\begin{aligned} \mathcal {L} (G, D) = \mathcal {L}_{\text {GAN}}(G, D) + \gamma \mathcal {L}_{1} (G) \end{aligned}$$

where $\gamma = 0.5$ balances the contributions of the adversarial and $L_1$ losses. The adversarial loss is:

2 $$\begin{aligned} \begin{aligned} \mathcal {L}_{\text {GAN}}(G, D)&= \frac{1}{N} \sum _{n=1}^{N} \mathbb {E}_{(X^{ar}_{n} \sim \mathcal {X}^{\text {ar}})}[\log D(X^{ar}_{n})] \\&+ \mathbb {E}_{(Z \sim N(0, 1), X_n^{\text {cyto}} \sim \mathcal {X}^{\text {cyto}})} [\log (1 - D(G(Z,X_n^{cyto})))] \end{aligned} \end{aligned}$$

The $L_1$ loss guides the learning process to produce outputs that are content-consistent with the inputs:

3 $$\begin{aligned} \mathcal {L}_{1}(G) = \frac{1}{N} \sum _{n=1}^{N} \mathbb {E}_{(X^{\text {ar}}_{n}\sim \mathcal {X}^{ar} ,Z \sim N(0, 1), X_n^{\text {cyto}} \sim \mathcal {X}^{\text {cyto}}) } [ \Vert X^{\text {ar}}_{n}- G(Z, X^{\text {cyto}}_{n}) \Vert _{1} ] \end{aligned}$$

#### Unconditional Multi-Area GAN

Previous research has shown a region-specific relationship between receptor densities and cytoarchitecture (e.g., Palomero-Gallagher et al., 2013; Zilles et al., 2002; Zilles & Amunts, 2015). As such, one would assume that the Unconditional multi-area GAN would be the suitable model for this study. Here, we would like to investigate the impact of area-specific conditioning. We train a Conditional single-area GAN on both V1 and M1 areas for each receptor. The architecture and loss functions remain unchanged from the Conditional single-area GAN.

### Evaluation Metrics

We evaluate our cGANs using five different metrics.

Mean Absolute Error (MAE; Rajkumar and Malathi (2016)) measures the pixel discrepancies between z-score normalized patch pairs $X^{\text {ar}}_{n}$ and $\hat{X}^{\text {ar}}_{n}$. The mean and standard deviation used to compute the z-scores for each patch are only computed for $X^{ar}$ and then applied to $\hat{X}^{ar}$.

Peak Signal-to-Noise Ratio (PSNR; Hore and Ziou (2010)) assesses the clarity of $\hat{X}^{\text {ar}}_{n}$ in comparison to $X^{\text {ar}}_{n}$. It is calculated as:

4 $$\begin{aligned} PSNR(X^{\text {ar}}_{n}, \hat{X}^{\text {ar}}_{n}) = 20 \log _{10}\left( \frac{MAX_{P}}{\sqrt{MSE(X^{\text {ar}}_{n}, \hat{X}^{\text {ar}}_{n})}}\right) \end{aligned}$$

where $MAX_{P}$ refers to the maximum possible pixel value which is 1 in our case.

Structural Similarity Index (SSIM; Ndajah et al. (2010)) is measured between $X^{\text {ar}}_{n}$ and $\hat{X}^{\text {ar}}_{n}$. It is defined as:

5 $$\begin{aligned} SSIM(X^{\text {ar}}_{n}, \hat{X}^{\text {ar}}_{n}) = \frac{(2\overline{X^{\text {ar}}_{n}}\overline{\hat{X}^{\text {ar}}_{n}} + c_1)(2\sigma _{X^{\text {ar}}_{n}\hat{X}^{\text {ar}}_{n}} + c_2)}{(\overline{X^{\text {ar}}_{n}}^2 + \overline{\hat{X}^{\text {ar}}_{n}}^2 + c_1)(\sigma _{X^{\text {ar}}_{n}}^2 + \sigma _{\hat{X}^{\text {ar}}_{n}}^2 + c_2)} \end{aligned}$$

where $\overline{X^{\text {ar}}_{n}}$, $\overline{\hat{X}^{\text {ar}}_{n}}$ are the mean pixel intensities; $\sigma _{X^{\text {ar}}_{n}}^2$, $\sigma _{\hat{X}^{\text {ar}}_{n}}^2$ are variances; $\sigma _{X^{\text {ar}}_{n}\hat{X}^{\text {ar}}_{n}}$ is standard deviation; $c_1$ and $c_2$ are stability constants.

The Fréchet Inception Distance (FID) (Yu et al., 2021) measures the similarity of feature distributions between $X^{\text {ar}}_{n}$ and $\hat{X}^{\text {ar}}_{n}$. This is achieved by using standard features from the Inception V3 network (Szegedy et al., 2016). The FID is calculated as follows:

6 $$\begin{aligned} FID(X^{\text {ar}}_{n}, \hat{X}^{\text {ar}}_{n}) = ||\mu _{X^{\text {ar}}_{n}} - \mu _{\hat{X}^{\text {ar}}_{n}}||^2 + \text {Tr}(\Sigma _{X^{\text {ar}}_{n}} + \Sigma _{\hat{X}^{\text {ar}}_{n}} - 2(\Sigma _{X^{\text {ar}}_{n}}\Sigma _{\hat{X}^{\text {ar}}_{n}})^{1/2}) \end{aligned}$$

### Experimental Setup

**Fig. 5:**
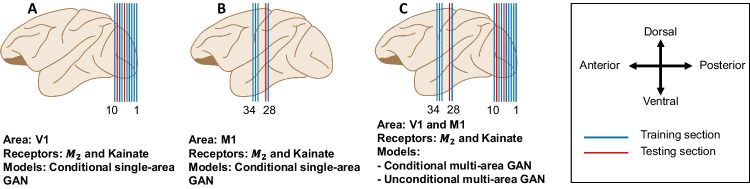
**A** and **B** show sections used by the conditional single-area GAN for training and testing for area V1 and M1, respectively, for both $M_2$, and Kainate receptors. **C** shows sections used by the Conditional and Unconditional multi-area GANs for both areas receptors

Training and testing splits for each cGAN model are outlined in Fig. 5. All models are trained for 250 epochs using Binary Cross Entropy loss and the Adam optimizer (Kingma & Ba, 2014), with a learning rate of 0.001. Betas *b*1 and *b*2 are set at 0.5 and 0.99, respectively. Gradient clipping is applied for training stability (Pascanu et al., 2013). Computations are performed on the JURECA DC supercomputer (Krause & Thörnig, 2018), utilizing four nodes each with four Nvidia A100 GPUs.

## Results

We evaluate the autoradiography patches generated by the proposed GAN models from quantitative and qualitative perspectives. The focus of our experiments is to verify that the generated distributions are representative for a given receptor and brain region, and that they are aligned with the cortical structure defined by the cell-body stained input patch.

### Quantitative Results

**Fig. 6:**
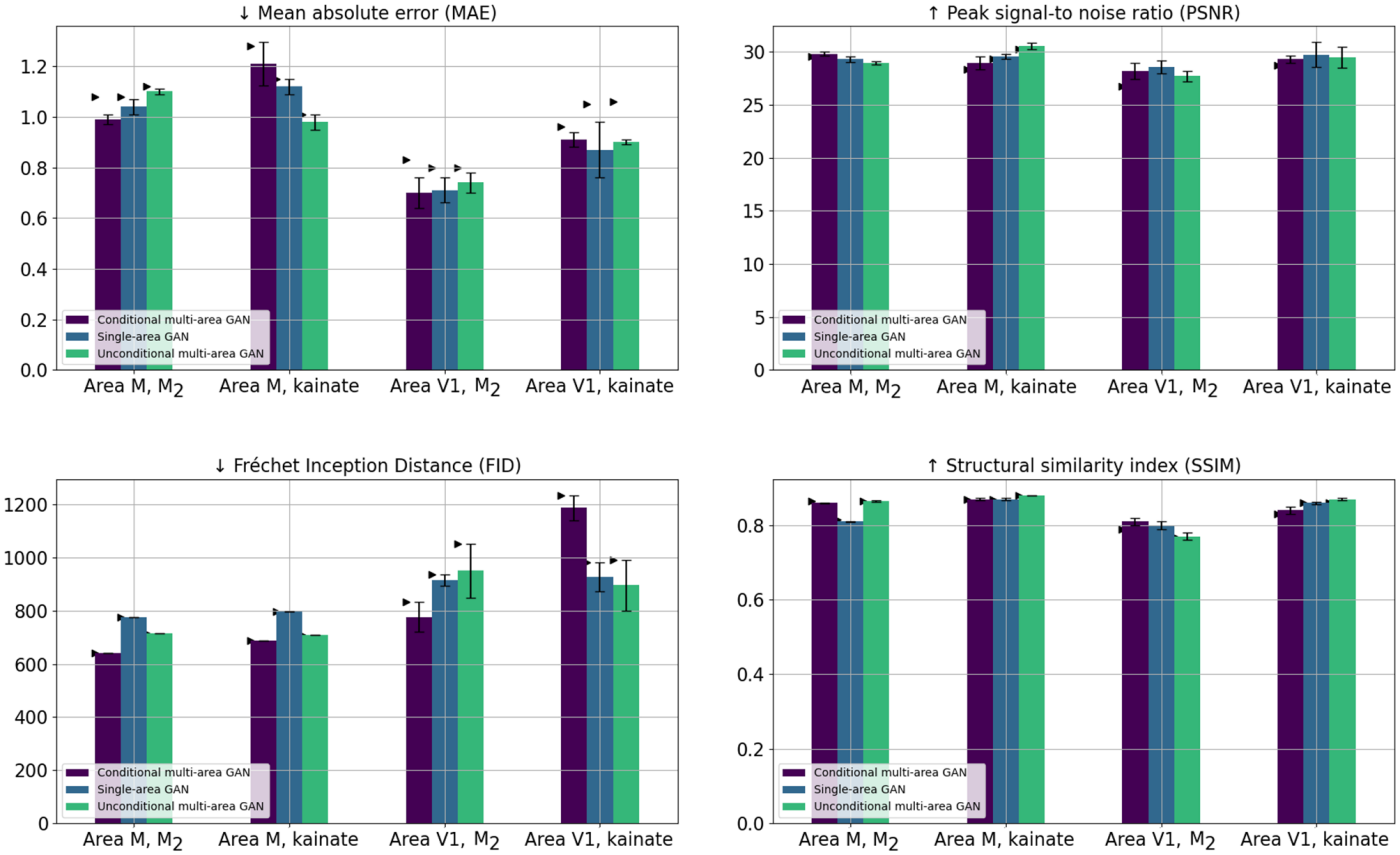
Metrics for the three different model variants across four experiments with test patches for different cortical brain areas (V1, M1) and receptors ($M_2$, kainate). Error bars refer to standard deviation, arrowhead markers indicate worst scores measured among all test patches. Left column: Smaller values imply better scores. Right column: Larger values imply better scores

Figure 6 presents mean values, standard deviations, and the least favorable patch scores-which are represented by either the minimum or maximum value, depending on the metric- for each of the metrics introduced in “Evaluation Metrics” section across four experiments that vary by brain region and receptor. Performance enhancement is signified by lower values for the MAE and FID scores, and by higher values for SSIM and PSNR

PSNR and SSIM scores are very similar for all three model variants (Fig. 6) in all studied brain regions. The z-score normalized MAE scores for area V1 exhibit an error margin equivalent to $80\%$ of 1 standard deviation from the target receptor patches, while for area M1, the error margin closely aligns with 1 standard deviation. SSIM values for all models and receptor-area pairings fall within the top $13\%$ of the SSIM scale (-1 to 1). FID scores vary by brain area. Models for area V1 have an average FID of 504.06, lower than those for area M1, which averages at 570.74.

Across all model variants, the reconstruction error as measured by MAE is significantly smaller for patches from the visual cortex compared to patches in the motor region. Additionally, FID and MAE show different results for patches with $M_2$ receptor distributions in area M1 compared to other area-receptor pairings. For example, while the unconditional multi-area GAN performs worst here according to MAE, it performs best in terms of FID. Such contradictory results between FID and MAE are not observed in other experiments.

### Qualitative Evaluation

**Fig. 7:**
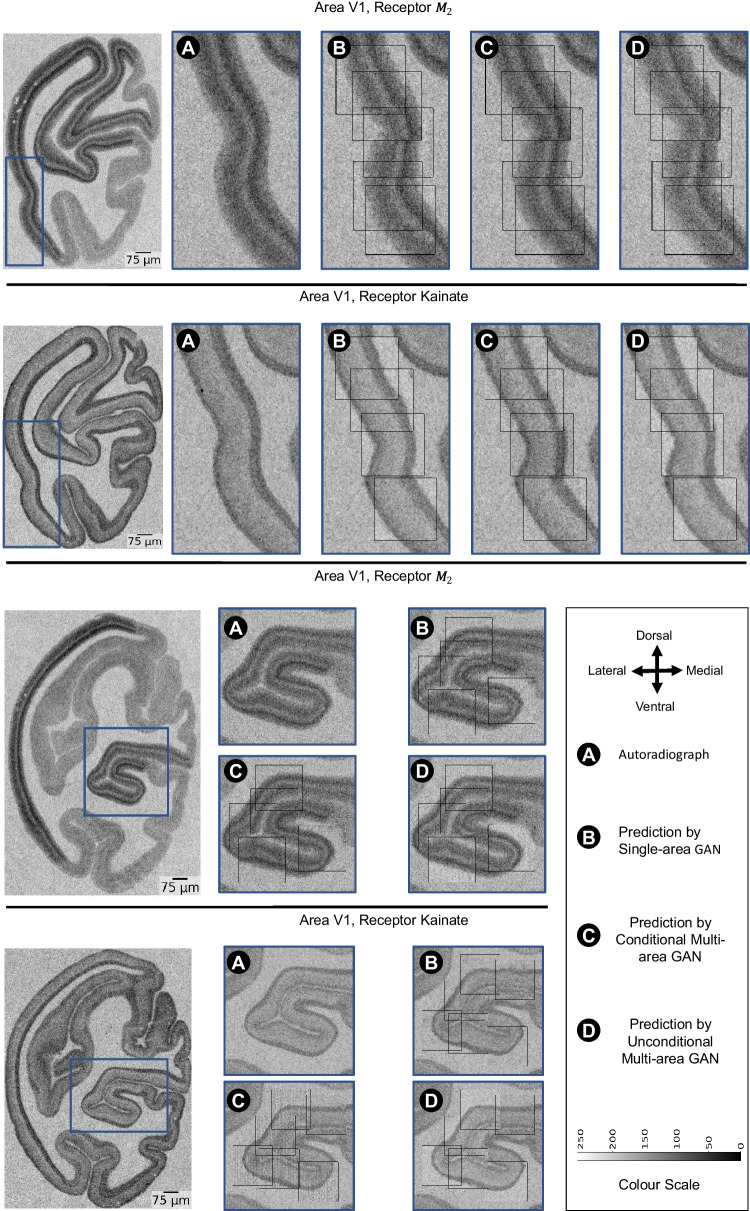
Comparison of the reconstructed $M_2$ and kainate receptor measures in area V1 across cGAN models. The figure presents mix-max contrast-enhanced reconstructions (for better readability) of $M_2$ and kainate receptor measures in area V1 using different trained cGANs. For each neurotransmitter receptor, the top section portrays a straight cortical morphology, while the bottom shows a sulcus

**Fig. 8:**
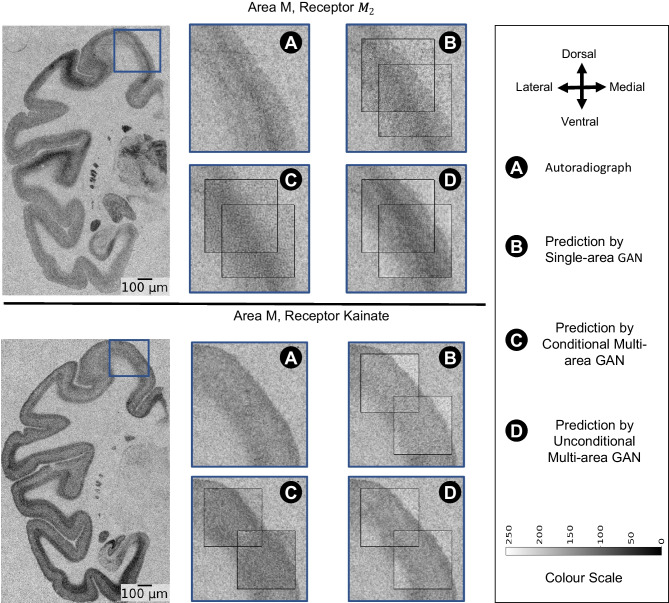
Comparison of the reconstructed kainate and $M_2$ receptor measures in area M1 across cGAN models. The figure presents min-max contrast-enhanced reconstructions (for better readability) of kainate and $M_2$ receptor measures in area V1 using different trained cGANs. The top section portrays the reconstruction of the $M_2$ receptor, while the bottom shows the reconstruction of the kainate receptor

For each model, we inpaint generated patches at their corresponding target positions in the autoradiography sections to visualize the consistency of generated receptor density distributions with the underyling cortical morphology. Figures 7 and 8 display aggregated patches for both receptors in areas V1 and M1, respectively. In order to maximize the visibility of receptor density pixel variations in the aforementioned figures, the inpainted patches were subjected to min-max contrast enhancement. This process adjusted each section’s pixel intensities to cover the entire intensity range observed in the section.

Figures 7 and 8 show that the models replicate target cortical orientations and curvatures across all receptor-area pairings. For instance, all model predictions for area V1 show that predicted receptor densities, for both receptors, are lower in the central layers of the cortex and increase towards the pial surface and grey/white matter boundary which conforms to the target receptor density in V1. For area M1, all models show higher kainate receptor density at the cortical edges, matching target densities. In contrast, with receptor $M_2$, densities are higher in the cortex center and lower at the edges. Aparently, the single-area GAN and conditional multi-area GAN preserve the contrast level quite closely, while the unconditional multi-area GAN displays a slightly lower contrast between the cortex center and edges in the shown example.

The predicted patches accurately reflect the thickness and laminar patterns of the target receptor patches. Nonetheless, in area V1, receptor $M_2$ patches exhibit a 10-pixel spatial shift, and in area M1, an 8-pixel spatial shift is observed across receptors. These spatial shifts, however, do not visually seem to impact the cortical thickness.

Additionally, patches from the unconditional multi-area GAN in area V1 display higher contrast than those from other models (see Fig. 7 panels (D)). Despite this higher contrast, visual inspection shows no significant alteration in the receptor density levels or in the laminar organization. In area M1, unconditional multi-area GAN does not replicate accurately the laminar structure of receptor $M_2$ despite scoring the highest SSIM and lowest FID values compared to the other models.

## Discussion

In this study, we applied three cGANs to predict $M_2$ as well as kainate distributions for areas V1 and M1 in the macaque monkey brain. We evaluated these cGANs both quantitatively using MAE, PSNR, FID, and SSIM, and qualitatively using visual assessment.

Our results demonstrate the capability of conditional GANs to generate realistic neurotransmitter receptor distributions as captured in autoradiography, as shown by MAE values ranging between 0.8 and 1.2 for all studied GAN model variants (Fig. 6). Such range is considered relatively low due to the noisy nature of the target autoradiography (see Fig. 1). As such, unlike area V1 (see Fig. 7), where patches show a well-differentiated laminar structure with sharp differences between the cortical layers, patches taken from area M1 display less pronounced laminar structure and are more noisy (see Fig. 8). This justifies that the average MAE across all models is higher for area M1 compared to area V1. Furthermore, the high range SSIM scores (see “Quantitative Results” section) highlights the models’ effectiveness in capturing textural similarities.

**Fig. 9:**
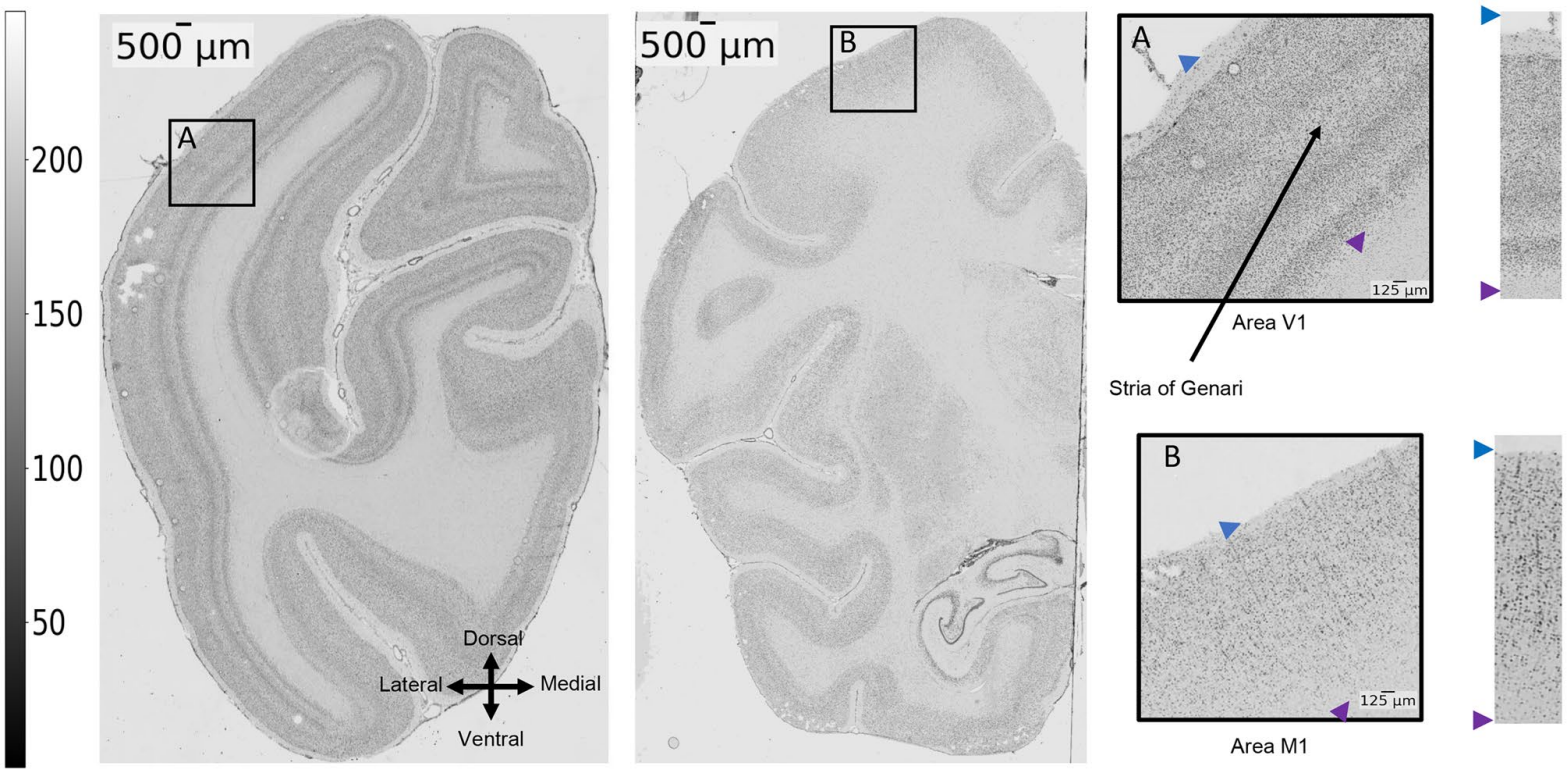
Illustration of laminar differences between area V1 and area M1. In contrast to the motor cortex, V1 exhibits a more distinct laminar structure; it shows a broad layer IV, where input arrives from the retina via the lateral geniculate body. The motor cortex is characterized by the presence of large pyramidal cells. Moreover, the delineation between the motor cortex and the white matter is less apparent due to reduced density in the lower layers in M1 as compared to V1. Note that the cell-body stained section shows a bubble artifact in the upper left part, which is located outside the cortical regions used in this study

A key aspect of our models is their ability to adequately replicate the cortical morphology of target receptor patches. All model variants receive a cortical image patch from a nearby cell-body stained section as an input, which they use to define the underlying cortical morphology (See Fig. 9). The overall morphology, such as curvature and laminar layer structure, are adequately preserved in the generated patches (Figs. 7 and 8).

However, generated patches occasionally suffer from systematic shifts (see Fig. 7 top panel, and Fig. 8 both panels), we expect to arise from remaining registration inaccuracies in the pairs of training patches. While there exist several automatic registration methods on scanned brain section data (Schubert et al., 2016; Modersitzki et al., 2001), the difference in resolution and pixel intensities makes these methods challenging to apply. Therefore, we resorted to manual registration which itself presented significant challenges due to the anatomical changes occurring between adjacent sections as well as the difference in resolution (i.e., a 1-pixel shift in the autoradiography results in a 20-pixel shift in the cell-body stained modality). Despite manual quality assessments and adjustments of the registration, there still exist a few errors which will be the subject of improvement in future studies. These errors could be also observed in the results in Fig. 6 showing that MAE value’s range is higher for predictions on area M1, compared to area V1. On the other hand, the observed variation in FID scores among receptors, particularly for receptor $M_2$ and in area M1, can be ascribed to the FID score computation method. This method utilizes a pre-trained Inception V3 network for feature extraction to quantify differences between predicted and target patches. Given that the Inception V3 network might be optimized for certain shapes and/or pixel intensities, it may inherently favor specific models or area-receptor pairings.

Besides the cortical morphology, a major challenge for image generation is the complex relationship between neurotransmitter densities and cytoarchitecture. The models cannot generally assume that a given distribution of cell bodies, as captured by the input image patches, implies a clearly defined neurotransmitter density distribution. Indeed, there is no consistent correlation between receptor densities and cell packing densities; furthermore, differences in receptor densities across layers do not trivially align with or predict layer-specific differences in cell packing densities (Zilles et al., 2004; Palomero-Gallagher & Zilles, 2019) (i.e., although it is true that most receptors for classical neurotransmitters are present at higher densities in the superficial than in the deeper cortical layers (Zilles & Palomero-Gallagher, 2017), it could also be shown that the cortical depths at which the borders between receptor architectonically defined layers occur within a given brain region do not coincide with those between the cytoarchitectonically identified layers of that area Palomero-Gallagher and Zilles (2019)). Furthermore, for any area throughout the cortical ribbon, a given layer can contain the highest density of one receptor type and the lowest of another type in that given area, and this not only holds true for isocortical but also for allocortical areas. E.g., Throughout the isocortex layer I contains one of the highest 5-HT1A receptor densities, but one of the lowest kainate receptor concentrations Zilles and Palomero-Gallagher (2017). Within the hippocampus, a prominent component of the allocortex, the pyramidal layer of the CA1-CA3 regions contains significantly higher AMPA and lower NMDA receptor densities than the adjacent layers (Palomero-Gallagher et al., 2003). We, therefore, expected a need to inform models about the brain region of each input patch and therefore chose three model variants for our experiments that differ substantially in the way they are informed about the brain region: by explicit conditioning, by separate region-specific training, and without conditioning. Contrary to the above mentioned-expectation, the unconditional multi-area GAN performed comparably to the remaining cGAN models in terms of replicating key visual cortical attributes (i.e., cortical thickness laminar definition) across various receptors and brain areas without specific brain area conditioning. This observation is supported by the close metric values and visual receptor quality shown in Figs. 7 and 8. A possible explanation for this behavior is that the networks have learned to derive distinct characteristics of cytoarchitecture from the input patches, which provides a sufficient prior and makes explicit conditioning or training unnecessary. However, we noticed that the unconditional multi-area GAN tends to preserve contrast slightly less accurately than the other models. A possible reason for this observation is the use of uncalibrated autoradiography scans, potentially making it difficult for the unconditional multi-area GAN to distinguish intensity balances in the different areas precisely. In future work, we aim to use calibrated autoradiography scans to avoid similar issues.

We want to stress that at the current stage of research, we do not consider the images generated by deep generative models as suitable to replace real histological observations. Generative models require careful interpretation and validation of their outputs, since their capacity allows them to add and remove information from the signal in complex ways, and the evaluation and interpretation of deep generative models is an active field of research. However, the proposed models provide potential for powerful data interpolation and imputation (Funck et al., 2022), and improved workflows for whole-brain 3D reconstruction and atlasing Clearly, interpolated data should always be clearly labeled and documented to enable proper interpretation and validation of derived data analysis results. Another important application of the proposed generative models is to help discover cross-modal relationships. For example, if a model can learn to predict one modality from another reliably, it provides indication for an underlying relationship that merits further investigation. This could help in the formulation of novel anatomical hypotheses and motivate the progression towards explainable AI models.

## Conclusion

We have demonstrated the application of cGANs for estimating neurotransmitter receptor densities ($M_2$ and kainate) in the macaque monkey’s primary visual (V1) and motor cortex (M1) from cell-body stained histology. The models were trained on aligned consecutive sections displaying cell-body stains and receptor distributions, effectively mapping between these two modalities. Qualitative and quantitative evaluations of our models underscore their ability to preserve cortical features such as laminar thickness and curvature, demonstrating the accuracy and reliability of the predicted receptor densities. This would allow the prediction of more region-based autoradiography data which could be used to refine the existing findings in brain receptors. Our models could also be useful to study the relationships between cytoarchitecture and receptor densities by attempting to analyze the feature maps and provide explainability for the prediction process. Additionally, our cGANs mark the first step towards receptor distribution prediction in the whole given brain area using only cell-body stained sections taken from an existent cytoarchitecture atlas.

## Data Availability

No datasets were generated or analysed during the current study.

## Code Availability

Code is available at: https://jugit.fz-juelich.de/a.nebli/gan-for-cyto2receptor-translation
